# Evaluation of Research Diagnostic Criteria in Craniofacial Microsomia

**DOI:** 10.1097/SCS.0000000000009446

**Published:** 2023-06-02

**Authors:** Ruben W. Renkema, V. de Vreugt, Carrie L. Heike, Bonnie L. Padwa, Christopher R. Forrest, David J. Dunaway, E.B. Wolvius, Cornelia J.J.M. Caron, Maarten J. Koudstaal

**Affiliations:** * The Dutch Craniofacial Center, Department of Oral and Maxillofacial Surgery, Erasmus University Medical Center, Sophia’s Children’s Hospital Rotterdam; † Seattle Children’s Craniofacial Center, Seattle, WA; ‡ The Craniofacial Centre, Boston Children’s Hospital, Boston, MA; § The Craniofacial Centre, the Hospital for Sick Kids, Toronto, Canada; ∥ The Craniofacial Unit, Great Ormond Street Hospital, London, UK

**Keywords:** Craniofacial microsomia, diagnosis, diagnostic criteria, goldenhar syndrome, hemifacial microsomia, oculo-auriculo-vertebral syndrome, sensitivity, validation

## Abstract

Characteristics of patients with craniofacial microsomia (CFM) vary in type and severity. The diagnosis is based on phenotypical assessment and no consensus on standardized clinical diagnostic criteria is available. The use of diagnostic criteria could improve research and communication among patients and healthcare professionals. Two sets of phenotypic criteria for research were independently developed and based on multidisciplinary consensus: the FACIAL and ICHOM criteria. This study aimed to assess the sensitivity of both criteria with an existing global multicenter database of patients with CFM and study the characteristics of patients that do not meet the criteria. A total of 730 patients with CFM from were included. Characteristics of the patients were extracted, and severity was graded using the O.M.E.N.S. and Pruzansky-Kaban classification. The sensitivity of the FACIAL and ICHOM was respectively 99.6% and 94.4%. The Cohen’s kappa of 0.38 indicated a fair agreement between both criteria. Patients that did not fulfill the FACIAL criteria had facial asymmetry without additional features. It can be concluded that the FACIAL and ICHOM criteria are accurate criteria to describe patients with CFM. Both criteria could be useful for future studies on CFM to create comparable and reproducible outcomes.

Craniofacial microsomia (CFM) is a clinical diagnosis based on the presence of facial features that are commonly associated with this congenital condition. This includes uni- or bilateral hypoplasia of facial structures related to the first and second pharyngeal arch, such as the mandible, orbit, ears, facial nerve, and soft tissue.^1–3^ The type and severity of affected structures varies largely among patients. Different diagnostic terms have been used to describe patients with these features, including Goldenhar syndrome, hemifacial microsomia, and oculo-auriculo-vertebral spectrum. Research has shown however that the phenotypes of patients who were diagnosed with these conditions do not meaningfully differ from those diagnosed with CFM.^4–6^ It remains debated in literature whether isolated microtia is a distinct entity or minor variant of CFM.^7–9^ The wide phenotypic and etiologic heterogeneity of CFM makes it difficult to establish standardized diagnostic criteria and evaluate treatment outcomes for large populations.^6,10^

Establishing diagnostic criteria can be used to improve clinical care to guide individual patients, improve communication among healthcare providers and set standards for research.^11^ Diagnostic criteria are a set of sings and/or symptoms that reflect the different features of any disease to accurately identify patients with the disorder.^11^ Such criteria are broad, to be able to cover the heterogeneity of clinical phenotypes. Nonetheless, development of such criteria in CFM is challenging due to the variation of clinical phenotypes, low prevalence and potential overlap with other craniofacial syndromes, such as Treacher Collins, Nager and CHARGE syndromes.

In recent years, 2 sets of phenotypic criteria for CFM have been developed for clinical research. Each set was developed independently and based on consensus among distinct multidisciplinary health care providers with expertise treating patients with CFM and researchers. The multicenter consortium ‘Facial Asymmetry Collaborative for Interdisciplinary Assessment and Learning (FACIAL)’, which started in 2009, is a network established to develop standardized definitions and study protocols to facilitate clinical research on CFM. This collaborative created eligibility diagnostic criteria for research based on the different CFM features.^12^ A similar initiative was done in 2017 by the ‘International Consortium for Health Outcomes Measurement (ICHOM)’, which aims to implement a global standard set to obtain comparable data for benchmarking and research.^13^

Comparison of these criteria might help implementation of the standards on a larger scale and improve comparison of research. This study aims to evaluate the FACIAL and ICHOM CFM criteria with an existing database of patients with CFM to research the sensitivity of the criteria and study the characteristics of CFM patients that do not reach the criteria.

## METHOD

A global multicenter database including patients with CFM diagnosed at the craniofacial centers of Erasmus University Hospital, Rotterdam, The Netherlands, Great Ormond Street Hospital, London, United Kingdom, Boston Children’s Hospital, Boston, U.S.A., and the Hospital for Sick Kids in Toronto, Canada was used for this study (Institutional Review Boards approval: Rotterdam: MEC-2012-248; London: 14 DS25; Boston: X05-08-058; Toronto: 1000053298). Patients who presented at 1 of the craniofacial units from January 1980 until January 2016 and received the clinical diagnosis CFM were included in the database, which was setup in 2016. Patients were identified using a search strategy on facial asymmetry, mandibular hypoplasia or microtia in the electronic patient management systems of all hospitals. In addition, all patients seen at the craniofacial outpatient clinics were checked to identify patients with CFM. Patients were included after they received the clinical diagnosis CFM after clinical assessment by an experienced craniofacial team followed by verification by peers (C.J.J.M.C. and B.P.) using clinical photographs, panoramic X-rays and/or computed tomography scans of the head. Patients with isolated anomalies, such as isolated microtia or isolated mandibular hypoplasia that did clinically not receive the diagnosis CFM were not included. Review of medical charts was performed and data on date of birth, sex, laterality and characteristics of facial features and extra craniofacial anomalies was extracted.

The type and severity of the affected tissues was scored according to the PAT-CFM as described by Birgfeld et al. which is based on the O.M.E.N.S.+ and Pruzansky-Kaban classification.^14–16^ This classification scores the degree of underdevelopment of the Orbit (O), Mandible (M), Ear (E), Facial Nerve (N) and Soft tissue (S) based clinical examination or facial photographs. The ‘+’ stands for the presence of extracraniofacial anomalies, including vertebral, cardiac or renal anomalies. The Pruzansky-Kaban classification is based on radiographic assessment and grades the severity of mandibular and temporomandibular joint hypoplasia in type I, IIA, IIB and III.^17–19^ Patients were considered eligible for this study if at least four items of the O.M.E.N.S. classification could be scored, in which the M score could be both the soft tissue PAT-CFM ‘M’ or the Pruzansky-Kaban classification.

The consensus-based diagnostic criteria for CFM that are examined were compiled by the FACIAL network and the ICHOM CFM group.^12,13^ The FACIAL criteria for CFM include one or more of the following diagnoses (Supplemental Table 1, Supplemental Digital Content 1, http://links.lww.com/SCS/F58): (1) microtia or anotia; (2) facial asymmetry and preauricular tag; (3) facial asymmetry and facial tag; (4) facial asymmetry and epibulbar dermoid; (5) facial asymmetry and lateral oral cleft (6) preauricular tag and epibulbar dermoid; (7) preauricular tag and lateral oral cleft; (8) facial tag and epibulbar dermoid; (9) lateral oral cleft and epibulbar dermoid. Facial asymmetry was in this study defined as skeletal hypoplasia, facial nerve deficit and/or soft tissue hypoplasia. Patients with other syndromic diagnosis or chromosomal abnormalities are excluded. The ICHOM CFM diagnostic criteria are based on a combination of 2 major criteria, or 1 major + 1 minor criteria, or 3+ minor criteria (Supplemental Table 2, Supplemental Digital Content 2, http://links.lww.com/SCS/F59).^13^ Major criteria are (1) mandibular hypoplasia; (2) microtia; (3) orbital/facial bone hypoplasia; (4) asymmetric facial movement. Minor criteria include (1) facial soft tissue deficiency; (2) preauricular tags; (3) lateral oral cleft; (4) clefting; (5) epibulbar dermoids; (6) hemivertebrae. Patients with other craniofacial syndromes or isolated typical Tessier clefting are also excluded in these criteria.

The main outcome of this study is to assess the sensitivity of both sets of CFM criteria (FACIAL and ICHOM) and the characteristics of patients who do not fulfil to either the FACIAL or ICHOM CFM criteria. The CFM criteria will be applied on the clinical characteristics according to the PAT-CFM of all patients with CFM included in our database. Patients with other craniofacial syndromes are excluded in both criteria and these were not included in the study.

### Statistical Analysis

Statistical analyses were performed using SPSS (2011, SPSS Inc., Chicago, IL, USA). Descriptive statistics were initially performed. A Cohen’s Kappa statistic was used to compare the ICHOM CFM diagnostic criteria to the FACIAL diagnostic criteria.^20^ This was interpreted following the guidelines of Landis and Koch.^20^ The effect of missing data on the outcomes was checked using multiple imputation analysis. If no effect was present, multiple imputation was not used.

## RESULTS

### Study Population

The clinical database included 730 patients with CFM (Supplemental Table 3, Supplemental Digital Content 3, http://links.lww.com/SCS/F60). Patients were diagnosed at the Boston Children’s Hospital (35%, n=253), Great Ormond Street Hospital London (34%, n=246), Erasmus Medical Center Rotterdam (22%, n=166), and The Hospital for Sick Kids Toronto (9%, n=65). It included more males (55%) than females (45%). Unilateral CFM (88%) was more common than bilateral CFM (12%). Patients with unilateral CFM had more right side (57%) than left side (43%) facial involvement. Among the patients with a skin tag (n=267), 216 (81%) patients had a facial tag, and 51 (19%) patients had a preauricular tag. Cleft palate was present in 98 (13%) patients, hemivertebrae in 65 (9%) patients, epibulbar dermoids in 84 (12%) patients and lateral oral cleft in 143 patients (20%).

### FACIAL Diagnostic Criteria

The FACIAL criteria were met by 689 patients, corresponding with a sensitivity of 94.4% A total of 41 patients did not meet these criteria and the false negative rate was 5.6% (Supplemental Table 4, Supplemental Digital Content 4, http://links.lww.com/SCS/F61). All patients that did not meet the FACIAL diagnostic criteria (n=41) had facial asymmetry without other additional features that are included in the FACIAL criteria. As displayed in Supplemental Table 5, Supplemental Digital Content 5, http://links.lww.com/SCS/F62 most patients fulfilled the FACIAL criteria based on the presence of microtia or anotia (89%). Ten percent of the patients (n=72) that met the FACIAL criteria did not have microtia or anotia. The presence of facial asymmetry with facial tags (31.3%) or with lateral oral cleft (20.8%) were other common characteristics to meet the FACIAL criteria, whereas 1.6% to 5.4% of the patients met the criteria without the presence of facial asymmetry (Supplemental Table 5, Supplemental Digital Content 5, http://links.lww.com/SCS/F62).

### ICHOM CFM Diagnostic Criteria

A total of 727 patients met the ICHOM CFM criteria and 3 patients with CFM did not. The ICHOM CFM diagnostic criteria had a sensitivity of 99.6% and a false negative rate of 0.4% (Supplemental Table 6, Supplemental Digital Content 6, http://links.lww.com/SCS/F63). Of the patients that met the ICHOM CFM diagnostic criteria, 667 patients (91.4%) had 2 major criteria, 669 patients (91.6%) 1 major and at least 1 minor, and 79 patients (10.8%) met the ICHOM criteria based on 3 or more minor criteria. Of the 79 patients with 3+ minor criteria, 68 patients (86.1%) had 2 major criteria as well and 77 patients (97.5%) had 1 major and 1 minor criterium.

The characteristic of the 3 patients that did not meet the ICHOM criteria are displayed in Supplemental Table 7, Supplemental Digital Content 7, http://links.lww.com/SCS/F64.

### Comparison Diagnostic Criteria

The Cohen’s kappa statistic to compare the ICHOM CFM criteria and the FACIAL CFM criteria was 0.38, indicating a fair agreement between both criteria. Multiple imputation of data showed no differences in outcome.

## DISCUSSION

This study aimed to research the sensitivity of the FACIAL and ICHOM criteria for CFM and study the characteristics of patients that did not meet the criteria. Both criteria show a high sensitivity (FACIAL 94.4% and ICHOM 99.6%) with a fair agreement between both criteria. In this studied cohort, the ICHOM criteria tend to be most accurate. All patients who did not meet the FACIAL criteria did have facial asymmetry without additional factors or microtia. Congenital facial hypoplasia with underdevelopment of one or more O.M.E.N.S. items without other additional anomalies could be identified as CFM. Those patients are not included as CFM by the FACIAL criteria.

Patients with isolated microtia were excluded in this study. In the FACIAL criteria, patients with isolated microtia are regarded to be part of the ‘CFM-spectrum’. Applying the FACIAL criteria would lead to a different cohort of CFM patients, possibly with a less severe phenotype as only the ears are affected. Those patients are missing in the CFM cohort studied in this study. The effect of including patients with microtia, who should be included according to the FACIAL criteria on the sensitivity of the ICHOM criteria could thus not be studied.

Both criteria were developed to study patients with CFM and compare outcomes. The usefulness of diagnostic criteria in CFM for clinical purposes is debatable. As CFM is heterogeneous, the treatment plan is based on individual needs and varies largely among patients. Also, there is overlap between other craniofacial conditions, e.g., Treacher Collins or Robin sequence, in which some aspects of the treatment plan during life might be similar. Therefore, it might be better to use eligibility criteria to study outcomes of treatment then diagnostic criteria. If the studied outcome is not dependent of a certain syndrome but of a specific characteristic, the studied cohort can be based on eligibility criteria rather than diagnostic criteria. By doing this, the outcomes are applicable to all patients with the defined criteria. Especially since most craniofacial syndromes show much overlap in their clinical presentation. Also, use of eligibility criteria could increase the sample size that can be studied, enhancing research on relatively rare craniofacial syndromes.

It is also questionable whether craniofacial microsomia is a true distinct entity. It is a syndrome with a specific phenotype as delineated in by the Pruzansky-Kaban and O.M.E.N.S. classification.^14,15,17^ CFM is heterogeneous, without showing clusters of specific patient groups.^6^ Some articles showed that CFM occurs more frequently in certain families, is related to specific pre-natal factors, or associated with genetic mutations.^21–25^ Nonetheless, the pathophysiology of CFM is yet unknown. Besides the facial anomalies, extra craniofacial anomalies might occur too.^26^ The heterogenic presentation, overlapping or possibly co-occurring with other syndromes might indicate that CFM is not a distinct entity but could be seen as a developmental disorder that constitute to a spectrum. This spectrum, varying in type and severity of affected structures, might include syndromes like the VACTERL association, limb-body wall complex or Mullerian duct aplasia, renal anomalies, cervicothoracic somite dysplasia (MURCS), and could be described as a “recurrent constellation of embryonic malformations” (RCEM).^27^ By abandoning the idea that CFM is a distinct entity but part of a spectrum with other developmental disorders, a RCEM, many more patients with overlapping features can be studied.^27,28^ This also advocates the use of eligibility criteria instead of diagnostic criteria.

There are some limitations in this study. An analysis on the specificity could not be performed as no control group with the characteristics of other craniofacial syndromes was included. The large cohort of CFM patients enabled us to study these criteria. Comparing the outcomes with other syndromes, which also creates the ability to identify diagnostic criteria using logistic regression, was not considered possible due to the high number of patients with other, rare, craniofacial syndromes that needed to be included.

Another consideration of this study is the included CFM cohort. All patients were identified using after a thorough search using search terms in all electronic patient management systems. After receiving the diagnosis CFM by an experienced craniofacial surgeon/team, the diagnosis was verified using radiographic or clinical images by peers. Nonetheless, no strict inclusion criteria were set-up to include the patients. As there is no ‘golden standard’ for CFM, the included cohort is based on an extensive approach to create a reproducible group of patients based on double checked clinical evaluation. By using this cohort, we can study whether the theoretically developed criteria match clinical patients with CFM, enabling future prospective research to include a well-defined cohort of patient with CFM.

Diagnostic criteria are set-up to score during consultation with the patient. Applying the criteria on retrospective data might be challenging as not all clinical characteristics are known. In our studied cohort patients were included from 1980 until 2016. Inclusion of older data could be challenging as more data might be missing. To encompass this difficulty in this retrospective analysis, only patients with at least 4 known items of the O.M.E.N.S. score were included. In additional, a multiple imputation analysis was used to score missing data. As this did not lead to any differences in outcome, the effect of missing data was considered neglectable.

It can be concluded that both the FACIAL and ICHOM criteria are useful criteria to describe patients with CFM with a high sensitivity and fair agreement between both criteria. The ICHOM criteria showed the highest sensitivity in this studied cohort. Both criteria are considered useful for future studies on CFM to create comparable and reproducible outcomes.

## Supplementary Material
