# Supplementary material for: Evaluation of Research Diagnostic Criteria in Craniofacial Microsomia
Source: J Craniofac Surg. 2023 Jun 2;34(6):1780–3. doi: 10.1097/SCS.0000000000009446 (PMC10445631; doi:10.1097/SCS.0000000000009446)
Supplement: Supplementary file 1 [file scs-34-1780-s001.docx]

**Supplemental Table 1.** FACIAL diagnostic criteria

| **FACIAL Inclusion criteria: (≥ 1 of the diagnoses below)** |
| --- |
| 1. Microtia |
| 1. Anotia |
| 1. Facial asymmetry + Preauricular tag |
| 1. Facial asymmetry + Facial tag |
| 1. Facial asymmetry + Epibulbar dermoid |
| 1. Facial asymmetry + Lateral oral cleft |
| 1. Preauricular tag + Epibulbar dermoid |
| 1. Preauricular tag + Lateral oral cleft |
| 1. Facial tag + Epibulbar dermoid |
| 1. Lateral oral cleft + Epibulbar dermoid |
| **Exclusion criteria:** |
| 1. Other syndromic diagnosis (e.g. Treacher Collins syndrome) with microtia and/or underdevelopment of the jaw |
| 1. Abnormal genetic studies |
