# Supplementary material for: Evaluation of Research Diagnostic Criteria in Craniofacial Microsomia
Source: J Craniofac Surg. 2023 Jun 2;34(6):1780–3. doi: 10.1097/SCS.0000000000009446 (PMC10445631; doi:10.1097/SCS.0000000000009446)
Supplement: Supplementary file 2 [file scs-34-1780-s002.docx]

**Supplemental Table 2.** ICHOM CFM diagnostic criteria

| **Conditions:** | **2 major criteria** | | **1 major + 1 minor criteria or** | | | **3+ minor criteria** | | |
| --- | --- | --- | --- | --- | --- | --- | --- | --- |
| **Major Criteria:** | Mandibular hypoplasia | Microtia | | Orbital/facial bone hypoplasia | Asymmetric facial movement | | | |
| **Minor criteria:** | Facial soft tissue deficiency | Pre-auricular tags | | Lateral oral cleft | Clefting | | Epibulbar dermoids | Hemivertebrae |
| **Exclusion criteria:** | Mandibulofacial dysostosis with microcephaly, Townes-Brocks Syndrome, Treacher Collins Syndrome, Auriculocondylar Syndrome, Bixler Syndrome, Branchiootorenal (BOR) Syndrome, CHARGE Syndrome, Miller Syndrome, Nager Syndrome, Oculoauriculofrontonasal Syndrome, Parry Rhomborg, Branchiooculofacial Syndromes (BOFS), isolated typical Tessier clefting (with no associated facial hypoplasia) | | | | | | | |
