# Supplementary material for: Evaluation of Research Diagnostic Criteria in Craniofacial Microsomia
Source: J Craniofac Surg. 2023 Jun 2;34(6):1780–3. doi: 10.1097/SCS.0000000000009446 (PMC10445631; doi:10.1097/SCS.0000000000009446)
Supplement: Supplementary file 3 [file scs-34-1780-s003.docx]

**Supplemental Table 3.** Characteristics of the complete study sample.

| Variable  N | Total study sample  (730) | FACIAL Criteria  (689) | ICHOM Criteria  (727) |
| --- | --- | --- | --- |
| Male  Female | 398 (54.5%)  332 (45.5%) | 374 (54.3%)  315 (45.7%) | 396 (54.5%)  331 (45.5%) |
| Bilateral CFM (yes) | 98 (12.3%) | 93 (12.9%) | 98 (12.6) |
| Unilateral CFM  Right side  Left side | 640 (87.7%)  364 (56.9%)  276 (43.1%) | 603 (87.5%)  345 (57.2%)  258 (42.8%) | 637 (87.6%)  361 (56.7%)  276 (43.3%) |
| Hemivertebrae (Yes) | 65 (8.9%) | 63 (9.1%) | 63 (8.9%) |
| Cleft Palate (Yes) | 98 (13.4%) | 90 (13.1%) | 98 (13.5%) |
| Skin tag  Pre auricular tag | 216 (29.6%)  51 (7.0%) | 216 (31.3%)  51 (7.4%) | 214 (29.4%)  51 (7.0%) |
| Epibulbair dermoid (yes) | 84 (11.5%) | 84 (12.2%) | 84 (11.6%) |
| Lateral oral cleft  Unilateral  Bilateral  Side unknown | 143 (19.6%)  105 (14.4%)  9 (1.2%)  29 (4.0%) | 143 (20.8%)  105 (15.2%)  9 (1.3%)  29 (4.2%) | 142 (19.5%)  104 (14.3%)  9 (1.2%)  29 (4.0%) |
| Mandibular hypoplasia (yes) | 705 (98.3%) | 664 (98.2%) | 703 (98.3%) |
| Microtia or anotia (yes) | 613 (85.6%) | 613 (89.5%) | 613 (86.0%) |
| Orbital bone hypoplasia (yes) | 342 (46.9%) | 323 (46.9%) | 342 (47.1%) |
| Asymmetric facial movement (yes) | 160 (43.1%) | 150 (43.7%) | 160 (43.4%) |
| Soft tissue deficiency (yes) | 612 (84.8%) | 578 (84.9%) | 611 (84.9%) |
