# Supplementary material for: Evaluation of Research Diagnostic Criteria in Craniofacial Microsomia
Source: J Craniofac Surg. 2023 Jun 2;34(6):1780–3. doi: 10.1097/SCS.0000000000009446 (PMC10445631; doi:10.1097/SCS.0000000000009446)
Supplement: Supplementary file 4 [file scs-34-1780-s004.docx]

**Supplemental Table 4.** Sensitivity and false negative rate FACIAL diagnostic criteria.

|  | CFM | No CFM |  |
| --- | --- | --- | --- |
| Patients that meet FACIAL criteria | 689 (94.4%) | 0 | 689 |
| Patients that do not meet FACIAL criteria | 41 (5.6%) | 0 | 41 |
|  | 730 (100%) | 0 | 730 |
