# Supplementary material for: Evaluation of Research Diagnostic Criteria in Craniofacial Microsomia
Source: J Craniofac Surg. 2023 Jun 2;34(6):1780–3. doi: 10.1097/SCS.0000000000009446 (PMC10445631; doi:10.1097/SCS.0000000000009446)
Supplement: Supplementary file 5 [file scs-34-1780-s005.docx]

**Supplemental Table 5.** Patients who did meet the FACIAL diagnostic criteria.

| Features | Patients (n=689) |
| --- | --- |
| Microtia or anotia | 613 (89.0%) |
| Facial asymmetry + Preauricular tag | 52 (7.5%) |
| Facial asymmetry + Facial tag | 216 (31.3%) |
| Facial asymmetry + Epibulbar dermoid | 84 (12.2%) |
| Facial asymmetry + Lateral oral cleft | 143 (20.8%) |
| Preauricular tag + Epibulbar dermoid | 11 (1.6%) |
| Preauricular tag + Lateral oral | 20 (2.9%) |
| Facial tag + Epibulbar dermoid | 37 (5.4%) |
| Lateral cleft + Epibulbar dermoid | 30 (4.4%) |
