# Supplementary material for: Evaluation of Research Diagnostic Criteria in Craniofacial Microsomia
Source: J Craniofac Surg. 2023 Jun 2;34(6):1780–3. doi: 10.1097/SCS.0000000000009446 (PMC10445631; doi:10.1097/SCS.0000000000009446)
Supplement: Supplementary file 6 [file scs-34-1780-s006.docx]

**Supplemental Table 6.** Sensitivity and false negative rate ICHOM CFM diagnostic criteria.

|  | CFM | No CFM |  |
| --- | --- | --- | --- |
| Patients that meet ICHOM criteria | 727 (99.6%) | 0 | 727 |
| Patients that do not meet ICHOM criteria | 3 (0.4%) | 0 | 3 |
|  | 730 (100%) | 0 | 730 |
