# Supplementary material for: Evaluation of Research Diagnostic Criteria in Craniofacial Microsomia
Source: J Craniofac Surg. 2023 Jun 2;34(6):1780–3. doi: 10.1097/SCS.0000000000009446 (PMC10445631; doi:10.1097/SCS.0000000000009446)
Supplement: Supplementary file 7 [file scs-34-1780-s007.docx]

**Supplemental Table 7.** Patients who did not meet the ICHOM CFM diagnostic criteria.

| Patient | 1 | 2 | 3 |
| --- | --- | --- | --- |
| Unilateral or bilateral CFM | Unilateral | Unilateral | Unilateral |
| Orbital hypoplasia | No | No | No |
| Mandibular hypoplasia | Yes | * | Yes |
| Microtia or anotia | No | No | No |
| Asymmetric facial movement | No | No | No |
| Soft tissue deficiency | * | Yes | No |
| Epibulbar dermoids | No | No | No |
| Lateral oral cleft | No | Yes | No |
| Cleft | No | No | No |
| Skin tags | Yes | Yes | No |
| Pre-auricular tags | No | No | No |
| Hemivertebrae | No | No | No |

*Unknown
